# TLR9 Inhibition Shortly After Mating Increases Fetal Resorption and Alters B- and T-Cell Costimulatory Phenotypes in an Abortion-Prone Mouse Model

**DOI:** 10.3390/ijms27020848

**Published:** 2026-01-14

**Authors:** Daria Lorek, Anna Ewa Kedzierska, Anna Slawek, Paulina Kubik, Anna Chelmonska-Soyta

**Affiliations:** 1 Hirszfeld Institute of Immunology and Experimental Therapy, Polish Academy of Sciences, 53-114 Wroclaw, Poland; anna.kedzierska@hirszfeld.pl (A.E.K.); anna.slawek@hirszfeld.pl (A.S.); paulina.kubik@hirszfeld.pl (P.K.); anna.chelmonska-soyta@hirszfeld.pl (A.C.-S.); 2 Department of Immunology, Pathophysiology and Veterinary Preventive Medicine, Wroclaw University of Environmental and Life Sciences, 50-375 Wroclaw, Poland

**Keywords:** pregnancy, TLR9, miscarriage, regulatory B cells, costimulatory molecules

## Abstract

Maternal immune tolerance and controlled inflammatory responses are essential for fetal development and successful pregnancy. Regulatory T cells (Tregs) and B cells with regulatory properties (Bregs) maintain this balance by limiting excessive immune activation through the secretion of anti-inflammatory and tolerogenic cytokines, such as IL-10, TGF-β, and IL-35. Moreover, alterations in the costimulatory potential of antigen-presenting cells (APCs), including B cells, modulate the activation and differentiation of T cells. Toll-like receptors (TLRs), particularly TLR9, influence B-cell antigen presentation and cytokine production, thereby affecting the balance between pro-inflammatory and tolerogenic responses at the maternal–fetal interface. TLR9 overexpression has been observed in several pregnancy-related disorders in both humans and murine models. In this study, we examine whether blocking TLR9 shortly after mating could improve pregnancy outcomes and modulate the regulatory and antigen-presenting functions of B cells, as well as their interactions with T cells. Using an abortion-prone murine model (CBA/J × DBA/2J), we show that intraperitoneal administration of a TLR9 antagonist (ODN 2088) shortly after mating increases embryo resorption in CBA/J females compared to controls without affecting implantation. Flow cytometry analysis further reveals that mice receiving the TLR9 antagonist are characterized by downregulation of CD80 and upregulation of CD86 on B cells, accompanied by reduced expression of CD40L and CD28 on T cells, as well as a lower percentage of Tregs and activated T cells. In conclusion, blocking TLR9 signaling shortly after mating does not improve pregnancy outcomes; conversely, it exacerbates pregnancy loss in the CBA/J × DBA/2J abortion-prone model, while altering the costimulatory phenotype of B and T cells and impairing Treg development during pregnancy.

## 1. Introduction

During pregnancy, the maternal immune system is challenged to balance protection against pathogens with tolerance to paternal antigens present in the developing fetus. Pregnancy is characterized by sequential shifts between pro-inflammatory and tolerogenic immune states. The early phase of pregnancy is dominated by pro-inflammatory responses, which are subsequently replaced by a tolerogenic milieu, followed again by a predominance of inflammation immediately prior to parturition. Thus, in early pregnancy, controlled inflammatory response is necessary for proper implantation, placental formation, and protection of both the mother and the fetus against pathogens. However, excessive or inappropriate inflammation can cause uterine and placental tissue damage, impair fetal development [1], and consequently increase the risk of complications such as spontaneous abortion [2], preterm labor [3,4], preeclampsia (PE) [5,6], and intrauterine growth restriction (IUGR) [7].

During pregnancy, disturbances in the immunological balance toward immune non-tolerogenic and pro-inflammatory activities can be caused not only by pathogens but also by non-infectious endogenous stressors released during tissue damage or cell death [8,9]. Both of these types of factors can activate pattern recognition receptors (PRRs), including Toll-like receptors (TLRs) [10], which initiate intracellular signaling pathways promoting the production of pro-inflammatory cytokines and other mediators [11].

Overexpression and excessive activation of TLRs has been observed in many pregnancy-related disorders in both humans and murine models [12]. Among these receptors, TLR9 is one of the most extensively studied in the context of both normal and complicated pregnancy. TLR9 recognizes not only unmethylated CpG motifs present in bacterial and viral DNA [13,14] but also endogenous DNA molecules released during pregnancy such as mitochondrial DNA (mtDNA) [15] and cell-free fetal DNA (cffDNA) [16].

Consistent with these findings, elevated TLR9 expression has been reported in the placental tissue [17,18,19] and peripheral blood plasmacytoid dendritic cells [20] of preeclamptic women, as well as in animal models of fetal growth restriction (FGR) [21], where elevated concentrations of released mtDNA and cffDNA have been demonstrated. Similar findings have been described in decidual tissue from women with spontaneous abortion and in female mice of the CBA/J × DBA/2J abortion-prone model [22]. Furthermore, increased TLR9 mRNA expression has been detected in the endometrium of women with recurrent implantation failure [23].

Moreover, experimental studies in murine and rat models have confirmed that excessive TLR9 activation may impair pregnancy, as stimulation with TLR9 agonists, such as CpG oligodeoxynucleotides (ODNs), cffDNA, or mtDNA was shown to induce IUGR [21], PE [17,24], fetal resorption [16,22,25,26,27], and preterm birth [25,27]. These effects were particularly pronounced in immunodeficient strains, such as non-obese diabetic (NOD) [27] or IL-10-deficient mice [25], indicating that impaired immune regulation exacerbates TLR9-driven pregnancy complications.

It has also been shown in the murine abortion-prone model (CBA/J × DBA/2J), as an established model of immunologically mediated pregnancy loss, that TLR9 activation contributes to increased fetal loss, likely due to a reduction in the frequency of regulatory T cells (Tregs) [22]—cells essential for maintaining maternal–fetal tolerance [28,29]. This indicates the engagement of TLR9 in the regulation of pregnancy-induced tolerance. Importantly, it is well-established that the activation of TLRs, including TLR9, can also influence the functioning of B cells by affecting both the production of pro- and anti-inflammatory cytokines and their activity as antigen-presenting cells (APCs) [30,31,32,33,34]. Regulatory B cells (Bregs), which secrete anti-inflammatory cytokines such as IL-10 [35], TGF-β [36], and IL-35 [37,38], cooperate with Tregs to maintain immune homeostasis [39]. In addition, as antigen-presenting cells, B cells modulate T-cell activation and shape adaptive immune responses [40].

Our previous studies support the hypothesis that B-cell-expressed TLR9 may be involved in the dysregulation of immune balance during pregnancy, as splenic B cells isolated during the pre-implantation period (day 3 of gestation) from abortion-prone females showed higher TLR9 mRNA expression but lower TLR9 protein levels compared with those from normal pregnancies [41]. This prompted us to consider B cells as important players in TLR9-mediated immune regulation in the context of early pregnancy.

Based on these findings, the aim of this study was to determine whether inhibition of TLR9 by the antagonist ODN 2088, administered immediately after mating, improves pregnancy outcomes in an abortion-prone murine model. In addition, we aimed to evaluate whether TLR9 blockade modulates the regulatory and antigen-presenting functions of B cells, as well as the expression of costimulatory molecules on T cells, in order to assess its impact on B–T cell interactions in a pregnancy model characterized by an increased risk of embryo resorption.

## 2. Results

### 2.1. TLR9 Inhibition Increased Fetal Resorption Without Affecting Implantation

**Figure 1:**
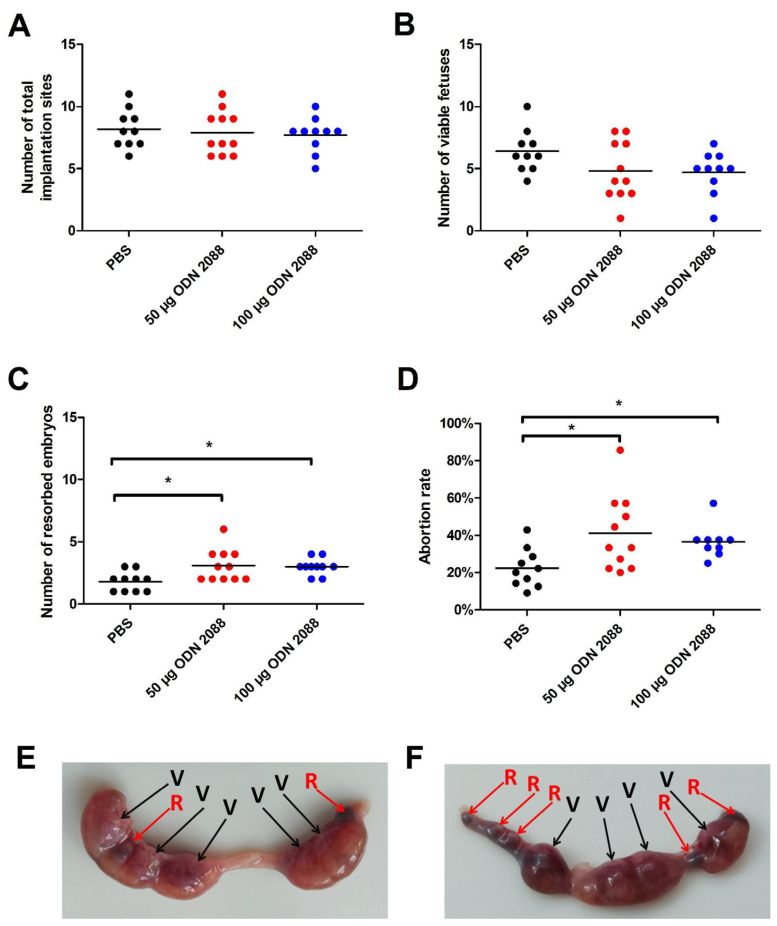
Effects of TLR9 inhibition on embryo viability and abortion rate in abortion-prone mice. (**A**) Total number of implantation sites, (**B**) viable fetuses, (**C**) resorbed embryos, and (**D**) abortion rate at 14 dpc in female abortion-prone mice after administration of 50 µg or 100 µg of the TLR9 antagonist ODN 2088 or vehicle (PBS). Panels (**E**,**F**) show representative uteri with viable fetuses (V) and resorbed embryos (R) from females injected with PBS (panel (**E**)) or ODN 2088 (panel (**F**)). Data are presented as individual values with the mean (n = 10–11). Statistical analyses were performed using one-way ANOVA with Dunnett’s multiple comparison post hoc test (for normally distributed data) or the Kruskal–Wallis test with Dunn’s multiple comparison post hoc test (for non-normally distributed data or those with non-equal variances). Normality was assessed using the Shapiro–Wilk test, and Bartlett’s test was performed to verify the homogeneity of variances between groups. * *p* < 0.05.

To assess the influence of TLR9 inhibition on pregnancy outcomes, viable fetuses and resorbed embryos were analyzed at 14 days post-coitum (dpc). We did not observe any changes in the total number of implantation sites between mice treated with the TLR9 antagonist ODN 2088 (50 µg and 100 µg) and those receiving only the vehicle phosphate-buffered saline (PBS) (Figure 1A). There were also no significant differences in the number of viable fetuses between the examined groups of pregnant females (Figure 1B). However, we found that administration of the TLR9 antagonist at doses of 50 µg and 100 µg significantly increased the number of resorbed embryos compared to that in the control group (Figure 1C).

At 14 dpc, the abortion rate was also calculated using the formula described in the Materials and Methods section. Female mice that received 50 µg or 100 µg of ODN 2088 demonstrated a higher abortion rate than those in the control group (Figure 1D).

Figure 1E,F show representative uteri with viable fetuses and resorbed embryos from females injected with PBS (Figure 1E) or ODN 2088 (Figure 1F).

### 2.2. TLR9 Inhibition Alters B-Cell Costimulatory Phenotype in Spleen and Para-Aortic Lymph Nodes Draining the Uterus

**Figure 2:**
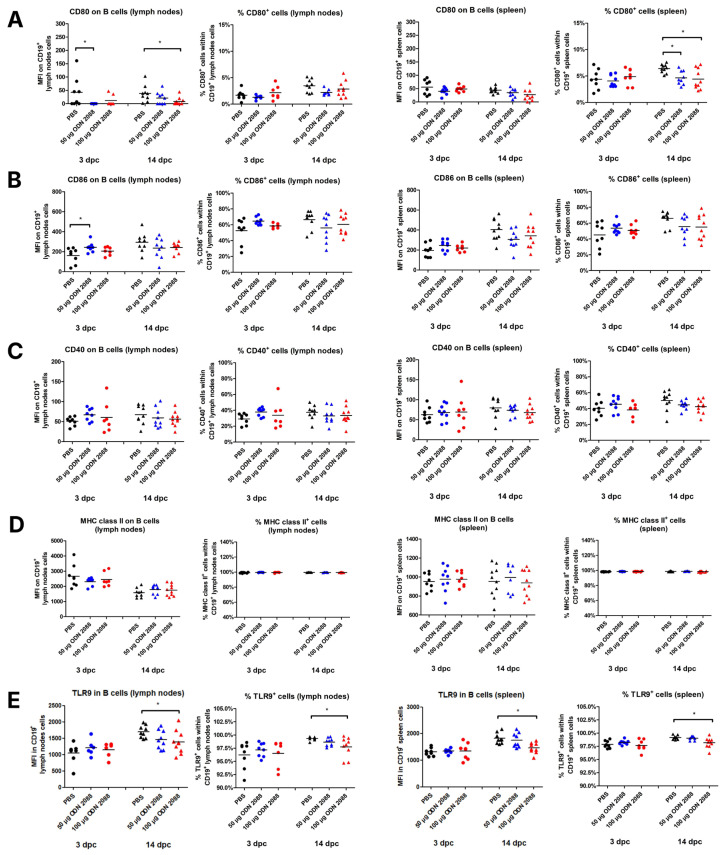
Expression of costimulatory molecules and MHC class II on B cells isolated from the spleen and para-aortic lymph nodes following TLR9 inhibition. Expression of (**A**) CD80, (**B**) CD86, (**C**) CD40, and (**D**) MHC class II on B cells (CD19^+^), and (**E**) TLR9 in B cells, as well as the percentages of (**A**) CD80^+^, (**B**) CD86^+^, (**C**) CD40^+^, (**D**) MHC class II^+^, and (**E**) TLR9^+^ cells within CD19^+^ cell populations in the spleen and lymph nodes from female abortion-prone mice. Comparisons at 3 and 14 dpc following the administration of 50 µg or 100 µg of the TLR9 antagonist ODN 2088 or vehicle (PBS). Data in the graphs are presented as individual values with the mean (n = 8–10). Statistical analyses were performed using one-way ANOVA with Dunnett’s multiple comparison post hoc test (for normally distributed data) or the Kruskal–Wallis test with Dunn’s multiple comparison post hoc test (for non-normally distributed data or those with non-equal variances). Normality was assessed using the Shapiro–Wilk test, and Bartlett’s test was performed to verify the homogeneity of variances between groups. * *p* < 0.05.

After administration of 50 µg ODN 2088, it was found that B cells in the para-aortic lymph nodes draining the uterus (PALNs) at 3 dpc showed reduced expression of CD80 molecules compared to control mice (Figure 2A). A similar decrease in CD80 expression was observed at 14 dpc in the same lymphoid organs following the treatment with the 100 µg dose. In splenic B cells, no differences in CD80 expression were detected at either 3 dpc or 14 dpc between the examined groups. However, a reduction in the proportion of CD80^+^ B cells was observed only at 14 dpc in the spleens of females injected with 50 or 100 µg of the TLR9 antagonist.

Analysis of CD86 expression on B cells revealed changes only at 3 dpc in the PALNs. At this time point, B cells from females treated with the TLR9 antagonist (50 µg ODN 2088) exhibited higher CD86 expression compared to those receiving the vehicle (Figure 2B). However, no changes were observed in the percentage of CD86^+^ cells within B-cell populations between the investigated groups in any lymphoid organ on any day of pregnancy.

The results indicated no significant differences in CD40 and MHC class II expression between ODN 2088-treated and control mice on B cells from PALNs and spleen, regardless of the day of pregnancy (Figure 2C,D). Similarly, there were no differences in the percentages of CD40^+^ and MHC class II^+^ cells within the CD19^+^ cell populations.

ODN 2088 administration did not cause changes in TLR9 expression in B lymphocytes in the spleen and lymph nodes on day 3 of pregnancy (Figure 2E). However, a decrease in the expression of this receptor in B cells derived from the spleen and lymph nodes was observed at 14 dpc in mice after administration of 100 µg of ODN 2088, when compared to the control group. Furthermore, a decreased frequency of B cells expressing TLR9 was observed in both the spleen and lymph nodes on day 14 of pregnancy following the administration of 100 µg ODN 2088. On day 3 of pregnancy, no differences in the frequency of CD19^+^TLR9^+^ cells among B lymphocytes derived from the examined organs were noted between the groups.

### 2.3. TLR9 Inhibition Does Not Alter Costimulatory Molecule or MHC Class II Expression on Uterine and Decidual B Cells, but It Changes TLR9 Expression Levels in Decidual B Cells

**Figure 3:**
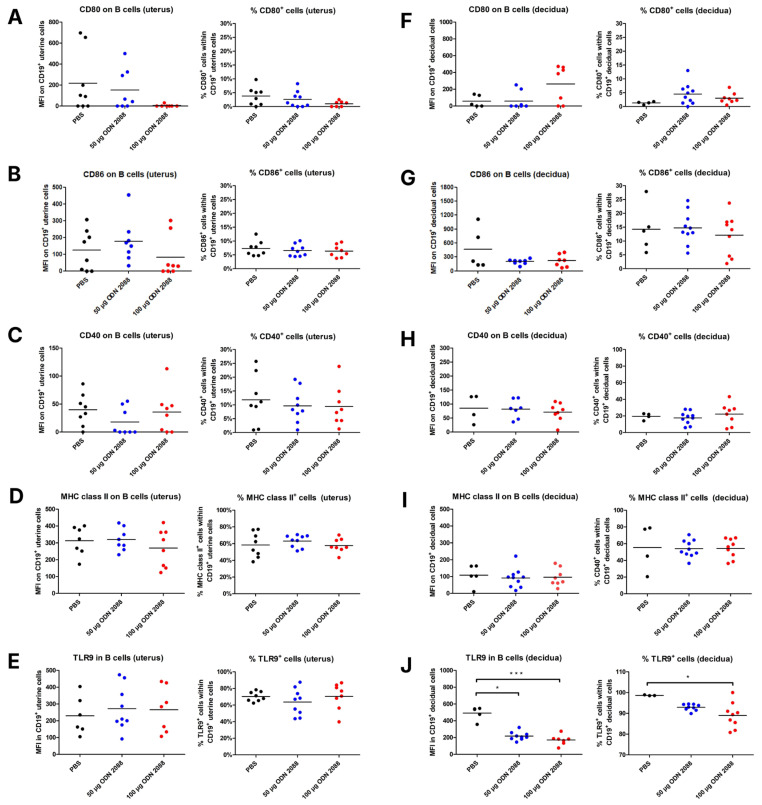
Expression of costimulatory molecules and MHC class II on B cells isolated from the uterus (3 dpc) and decidua (14 dpc) following TLR9 inhibition. Expression of (**A**,**F**) CD80, (**B**,**G**) CD86, (**C**,**H**) CD40, and (**D**,**I**) MHC class II on B cells (CD19^+^ cells), and (**E**,**J**) TLR9 in B cells, as well as the percentages of (**A**,**F**) CD80^+^, (**B**,**G**) CD86^+^, (**C**,**H**) CD40^+^, (**D**,**I**) MHC class II^+^, and (**E**,**J**) TLR9^+^ cells within uterine (3 dpc) or decidual (14 dpc) CD19^+^ cells from female abortion-prone mice following the administration of 50 µg or 100 µg of the TLR9 antagonist ODN 2088 or vehicle (PBS). Data in the graphs are presented as individual values with the mean (n = 4–9). Statistical analyses were performed using one-way ANOVA with Dunnett’s multiple comparison post hoc test (for normally distributed data) or the Kruskal–Wallis test with Dunn’s multiple comparison post hoc test (for non-normally distributed data or those with non-equal variances). Normality was assessed using the Shapiro–Wilk test, and Bartlett’s test was performed to verify the homogeneity of variances between groups. * *p* < 0.05; *** *p* < 0.001.

Analyzing the expression of selected antigens on B lymphocytes in the uterus on day 3 of pregnancy, no significant differences were observed in their expression between the group of mice that received the TLR9 antagonist at doses of 50 µg or 100 µg and the control group. Additionally, the frequency of B lymphocytes with the phenotypes CD19^+^CD80^+^, CD19^+^CD86^+^, CD19^+^CD40^+^, CD19^+^MHC II^+^, and CD19^+^TLR9^+^ in the uterus did not differ between mice that received ODN 2088 (at doses of 50 µg or 100 µg) and those administered PBS (Figure 3A–E).

However, on day 14 of pregnancy, B lymphocytes in the decidua exhibited lower TLR9 expression in mice that received ODN 2088 at both 50 µg and 100 µg doses, when compared to B cells from the control group (Figure 3J). Moreover, the frequency of decidual B lymphocytes expressing TLR9 was reduced in mice injected with 100 µg ODN 2088, compared to that in the control group (Figure 3J).

The expression of the other analyzed antigens on decidual B lymphocytes, as well as the frequency of B cells expressing these antigens, did not differ between the groups of mice (Figure 3F–I).

### 2.4. TLR9 Inhibition Lowers T-Cell Costimulatory Receptor Expression in Spleen and Para-Aortic Lymph Nodes Draining the Uterus

**Figure 4:**
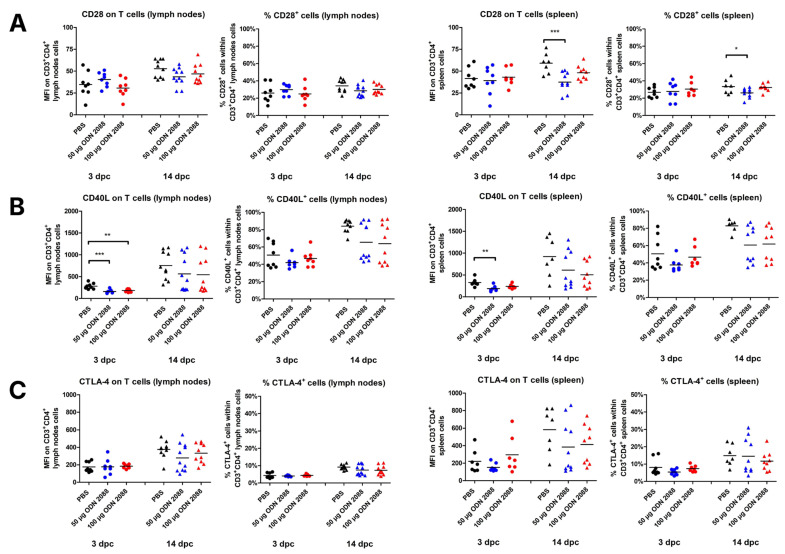
Expression of ligands for costimulatory molecules on T cells isolated from the spleen and para-aortic lymph nodes following TLR9 inhibition. Expression of (**A**) CD28, (**B**) CD40L, and (**C**) CTLA-4 on T cells (CD3^+^CD4^+^ cells), as well as the percentages of (**A**) CD28^+^, (**B**) CD40L^+^, and (**C**) CTLA-4^+^ cells within T cells in the spleen and lymph nodes from female abortion-prone mice. Comparisons at 3 and 14 dpc after the administration of 50 µg or 100 µg of the TLR9 antagonist ODN 2088 or vehicle (PBS). Data in the graphs are presented as individual values with the mean (n = 8–10). Statistical analyses were performed using one-way ANOVA with Dunnett’s multiple comparison post hoc test (for normally distributed data) or the Kruskal–Wallis test with Dunn’s multiple comparison post hoc test (for non-normally distributed data or those with non-equal variances). Normality was assessed using the Shapiro–Wilk test, and Bartlett’s test was performed to verify the homogeneity of variances between groups. * *p* < 0.05; ** *p* < 0.01; *** *p* < 0.001.

The data indicated that CBA/J females, after administration of ODN 2088 (dose of 50 µg), presented lower expression of CD28 molecules on T cells derived from the spleen and a lower percentage of CD28^+^ splenic CD3^+^CD4^+^ cells at 14 dpc than control mice (Figure 4A). No changes were observed in the expression of CD28 receptors on T cells or in the percentage of CD28^+^ cells within CD3^+^CD4^+^ cell populations in the PALNs at 14 dpc and in both the PALNs and spleen at 3 dpc.

The study showed that CD40L was downregulated at 3 dpc on T cells in the spleen (ODN 2088, dose 50 µg) and PALNs (ODN 2088, dose 50 µg and 100 µg) of mice treated with the TLR9 antagonist (Figure 4B). At 14 dpc, no differences in CD40L expression on T cells were observed between the groups in either the spleen or PALNs. There were also no changes in the percentages of CD40L^+^ cells within T cells from the spleen and PALNs (at 3 and 14 dpc) between ODN 2088- and vehicle-treated mice.

Administration of the TLR9 antagonist did not affect the expression of CTLA-4 on T cells in the spleen or PALNs at 3 and 14 dpc (Figure 4C). No changes in the proportion of CTLA-4^+^ T cells were observed between the examined groups of pregnant females at either time point or in either lymphoid organ.

### 2.5. TLR9 Inhibition Did Not Affect Breg Frequency and B-Cell IL-10/IL-6 Expression in Spleen and Para-Aortic Lymph Nodes Draining the Uterus

**Figure 5:**
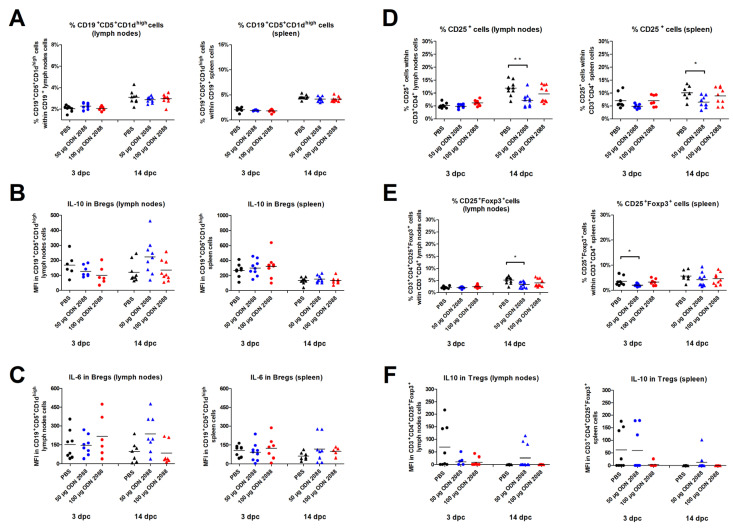
Frequency of regulatory B and T cells and cytokine expression by these cells following TLR9 inhibition. (**A**) Percentage of Bregs (CD19^+^CD5^+^CD1d^high^ cells) within B cells, (**D**) CD3^+^CD4^+^CD25^+^ cells, and (**E**) Tregs (CD3^+^CD4^+^ CD25^+^ FoxP3^+^) within T cells, as well as the expression levels of IL-10 in (**B**) Bregs and (**F**) Tregs, and IL-6 in (**C**) Bregs in the spleen and lymph nodes from female abortion-prone mice. Comparisons at 3 and 14 dpc after the administration of 50 µg or 100 µg of the TLR9 antagonist ODN 2088 or vehicle (PBS). Data are presented as individual values with the mean (n = 8–10). Statistical analyses were performed using one-way ANOVA with Dunnett’s multiple comparison post hoc test (for normally distributed data) or the Kruskal–Wallis test with Dunn’s multiple comparison post hoc test (for non-normally distributed data or those with non-equal variances). Normality was assessed using the Shapiro–Wilk test, and Bartlett’s test was performed to verify the homogeneity of variances between groups. * *p* < 0.05; ** *p* < 0.01.

Administration of ODN 2088 did not alter the proportion of Bregs (CD19^+^CD5^+^CD1d^high^ cells) within the CD19^+^ cell populations derived from the spleen and PALNs at 3 and 14 dpc (Figure 5A). The expression level of IL-10 in Bregs was also similar between the examined groups of mice in both the spleen and PALNs at these time points (Figure 5B).

TLR signaling is known to be involved in the production of pro-inflammatory cytokines by B cells. This phenomenon has also been observed in the population of B cells suspected to be Bregs [42,43]. Therefore, in this experiment, the expression level of IL-6 in Bregs was also examined. However, the IL-6 expression of B cells derived from the spleen and PALNs at 3 and 14 dpc remained at the same level between the ODN 2088-treated and vehicle-treated female mice (Figure 5C).

### 2.6. TLR9 Inhibition Decreased the Percentage of T Regulatory Cells in the Spleen and Para-Aortic Lymph Nodes Draining the Uterus

CBA/J females injected with 50 µg of ODN 2088 showed a lower percentage of CD25^+^ cells within T-cell populations in the spleen and PALNs at 14 dpc compared to control mice (Figure 5D). At 3 dpc, the proportion of CD25^+^ cells among CD3^+^CD4^+^ cell populations obtained from the spleen and PALNs was similar among the analyzed groups.

The results also revealed that, following administration of the TLR9 antagonist at a dose of 50 µg, females had a lower proportion of CD3^+^CD4^+^CD25^+^Foxp3^+^ (Tregs) within T-cell populations in the spleen at 3 dpc and in the PALNs at 14 dpc (Figure 5E). At 3 dpc in the PALNs and 14 dpc in the spleen, there were no significant changes in the percentage of Tregs within CD3^+^CD4^+^ cell populations between ODN 2088- and vehicle-treated mice.

Furthermore, the expression of IL-10 by CD3^+^CD4^+^CD25^+^Foxp3^+^ cells did not differ significantly between the groups (Figure 5F).

## 3. Discussion

Maternal and fetal immune tolerance depends on precise regulation of interactions between cells of the innate and adaptive immune systems, including antigen-presenting cells and specific regulatory lymphocytes (Tregs and Bregs). This regulation is particularly important during the peri-implantation period, as immune activation must be tightly controlled to enable implantation and establish tolerance to paternal antigens. Disturbances in this balance, resulting from the excessive activation of PRRs (including TLRs), have been linked to pregnancy complications.

The CBA/J × DBA/2J mice model, which is prone to miscarriage, provides a clear example of how immune dysregulation in early pregnancy impacts pregnancy outcomes. In this model, impaired immune regulation is caused by enhanced alloantigen recognition, excessive activation of effector T cells, a predominance of Th1 over Th2 responses, and a diminished pool of regulatory T and B lymphocytes, ultimately leading to fetal resorption [44,45,46]. Both suppression of inflammatory processes [47,48,49,50,51,52,53,54] and an increase in the number of regulatory cells (Tregs and Bregs) [44,46,55,56,57] have been shown to reduce the percentage of resorbed embryos. A decrease in resorption rates has also been observed under conditions of reduced activity of antigen-presenting cells, for example, when blocking costimulatory molecules such as CD80 and CD80/CD86 [48,49]. Since both the regulatory functions of immune cells and their activity as APCs are dependent on TLR signaling, these receptors may play a key role in maintaining immune balance in pregnancy. Because B lymphocytes perform both functions—acting as antigen-presenting cells and as regulatory B cells secreting IL-10—their activity is dependent on the proper activation of TLRs, making activation of TLRs in B cells particularly important for establishing early immunotolerance.

Therefore, this study aimed to determine whether inhibition of TLR9 activity via administering the antagonist ODN 2088 at the time of conception could modulate immune regulation and improve pregnancy outcomes in the murine abortion-prone model CBA/J × DBA/2J.

The results demonstrated that TLR9 inhibition in abortion-prone mice was not protective but, to the contrary, contributed to an increase in embryo resorption. Previous studies conducted in this murine model [22,58], in other models with impaired immune regulation (NOD mice and IL-10^−/−^ mice) [25,27], and in normal pregnancies [16,26] have shown that enhanced TLR9 signaling increases the risk of miscarriage. Our study provides the first evidence that the inhibition of TLR9 activity at the time of conception in abortion-prone mice is also detrimental.

Furthermore, we observed that ODN 2088 administration did not significantly affect the number of implantation sites, suggesting that early TLR9 signaling in the considered abortion-prone murine model may not play a critical role in blastocyst attachment or endometrial receptivity. However, the increased rate of fetal resorption observed later in gestation indicates dysregulation of the immune regulatory mechanisms required to maintain pregnancy rather than those involved in initiating it. To better understand the processes underlying this observation, we analyzed B cells with respect to their regulatory functions and antigen-presenting capacity, which can influence the induction and activity of Tregs.

In our experiment, we observed that the inhibition of TLR9 signaling after mating led to changes in the costimulatory phenotype of B- and T-cells (summarized in Table A1 and Table A2). After the administration of ODN 2088, a decrease in CD80 and an increase in CD86 on B cells were observed, along with a reduction in CD28 expression on T cells, indicating potential alterations in the interactions between these cells. Moreover, T cells from females with inhibited TLR9 signaling after mating were characterized by lower CD40L expression. The CD40–CD40L interaction between T and B cells is well understood and plays a crucial role in regulating the immune response [59]. CD40 is expressed on APCs, including B cells [60], whereas its ligand, CD40L, is found on activated T cells, with expression levels increasing following T-cell receptor (TCR) stimulation [61]. Stimulation of CD40 on B cells is crucial for their specific activation, initiation of the humoral response, and generation of memory B cells. In both humans and mice, CD80 and CD86 exhibit increased expression after CD40 activation, enhancing the efficiency of B cells as APCs [59]. Conversely, the interaction of CD28 on T cells with CD80/CD86 enhances CD40L expression, indicating a positive feedback loop involving CD40 on B cells and CD28 on T cells [62,63]. The lower expression of CD40L on T cells observed in the present study after the administration of the TLR9 antagonist may indicate weaker activation of these cells during the examined days of pregnancy. Consequently, this could result in a lower frequency of activated T cells (CD3^+^CD4^+^CD25^+^) as well as Tregs (CD3^+^CD4^+^CD25^+^FoxP3^+^), as observed in ODN 2088-treated females compared to controls (summarized in Table A3).

It has been demonstrated that Tregs require signaling between CD28 and CD86 for survival, proliferation, and activation [64]. B cells from females treated with ODN 2088 exhibited higher CD86 expression, suggesting an enhanced ability to interact with CD28, leading to stronger activation of both T cells and Tregs. It should also be noted that other APCs, such as dendritic cells and macrophages, also express TLR9 [65,66]. Therefore, administration of ODN 2088 may have reduced the antigen-presenting capacity of the other APCs, potentially affecting both the number and activation of Tregs. For this reason, future studies should consider examining these populations in more detail.

Besides Tregs, another important group of suppressor cells are Bregs. In the considered abortion-prone pregnancy model, it has been shown that Bregs with the phenotype CD19^+^CD5^+^CD1d^high^ may participate in the regulation of immune tolerance [46,67]. Therefore, in the present study, this population of B cells was selected to assess the impact of TLR9 inhibition on Breg frequency (summarized in Table A3). Since Bregs are characterized by IL-10 expression but can simultaneously produce pro-inflammatory cytokines such as IL-6, which may counteract their suppressive functions [42,68], both IL-10 and IL-6 expression levels were analyzed. However, no statistically significant differences in cytokine expression were observed between the groups. These results suggest a probable lack of direct or indirect effects of TLR9-signaling inhibition shortly after fertilization on the frequency of Bregs and their expression of the examined cytokines in the abortion-prone mouse model during pregnancy.

We also examined TLR9 expression in B cells isolated from female mice after administering ODN 2088. Interestingly, changes in the expression of this receptor in these cells between females that received the TLR9 antagonist and the control group were noticeable on the 14th day of pregnancy but not on the 3rd day. This suggests long-term effects of ODN 2088. Studies have shown that ODN 2088 inhibits the binding of both natural and synthetic ligands to TLR9 [69,70] and that its effect on B cells is manifested by impaired proliferation, reduced viability, and reduced IL-6 production, persisting for several hours after activation with specific agonists [69]. In most studies, ODN 2088 is used primarily to block signaling pathways, without detailed analysis of the kinetics of changes in the expression of the receptor itself. Therefore, it is currently unclear how rapidly TLR9 expression may change in B cells following exposure to this antagonist. Furthermore, there is a lack of in vivo pharmacodynamic data specifically for the ODN 2088 molecule, making it difficult to determine its persistence in the organism and the duration of its inhibitory effect following intraperitoneal (i.p.) administration.

Although our study provides new insights into the effects of TLR9 antagonists during pregnancy, several limitations should be considered when interpreting the results. First, we focused on the CBA/J × DBA/2J abortion-prone model, a well-established system for studying immune-mediated pregnancy disorders, which reproduces some immune abnormalities observed in women with recurrent pregnancy loss, including enhanced inflammatory responses and impaired immune tolerance [71]. However, this model does not capture other causes of pregnancy loss, such as hormonal or non-immunological factors, and therefore, our findings should be interpreted within the immunological context. Studies using additional pregnancy models could provide a broader biological perspective and allow analysis of other influences on TLR9 signaling and its inhibition during pregnancy. Although the available data remain inconsistent [23], changes in TLR9 expression and modifications of TLR9 signaling have been reported both in early pregnancy in animals [72] and across the menstrual cycle in humans [73,74], suggesting that regulation of this pathway may also depend on the hormonal context. Second, only selected immune cell populations were analyzed. It should be emphasized that intraperitoneal administration of a TLR9 inhibitor has a systemic effect; moreover, TLR9 is expressed not only in immune cells (e.g., dendritic cells and macrophages) [75,76] but also in non-immune cell types, including trophoblast, epithelial, and endothelial cells [18,70,77,78]. Thus, the observed changes in B and T cells may reflect both the direct effects of TLR9 inhibition and indirect modulation through other cell populations. Third, in our study, we demonstrated that ODN 2088 significantly influenced the expression of costimulatory molecules as well as TLR9 by B cells. Thus, while our findings suggest functional effects, they do not fully clarify the relationship between receptor expression and downstream signaling, such as MyD88 and other proteins in the pathway. Previous studies in sheep have shown variable patterns of TLR9 and MyD88 expression: Wu et al. [72] reported that during early pregnancy, TLR9 levels decreased while MyD88 and TRAF6 levels remained unchanged or increased in lymph nodes, whereas other studies found that in the spleen, TLR9 expression increased in parallel with MyD88 [79]. Additionally, TLR9 signaling can occur independently of MyD88 [80,81]. Therefore, a lack of direct correlation between TLR9 expression and the expression of signal transduction-related proteins complicates the interpretation of cause–effect relationships. Despite these limitations, our results indicate the inhibitory effectiveness of ODN 2088 in blocking TLR9 receptor activation on functional costimulatory responses and TLR9 expression. Further research is needed to clarify these complex interactions during pregnancy.

**Figure 6:**
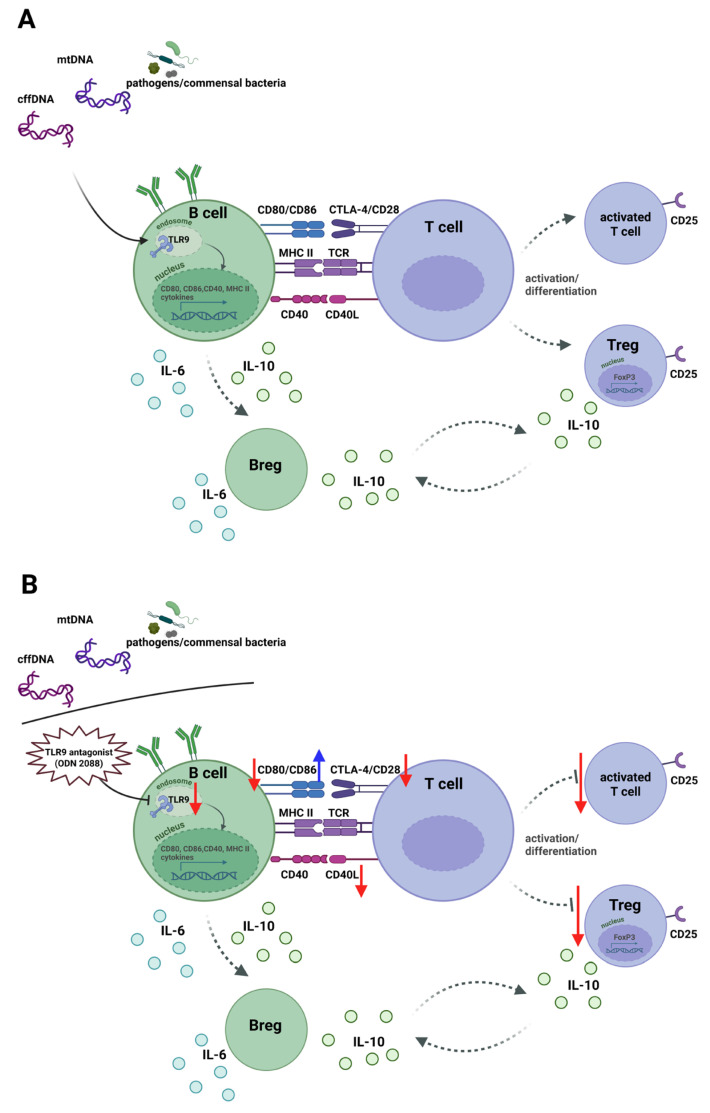
Schematic of the effects of TLR9 activation and inhibition in B cells on T-cell responses under steady-state conditions and following treatment with a TLR9 antagonist during early pregnancy in females in the murine abortion-prone model. (**A**) Endogenous or pathogens DNA activates TLR9 in B cells, inducing CD80, CD86, CD40, and MHC class II expression for antigen presentation. MHC class II–TCR binding provides the first signal, and CD80/CD86-CD28 and CD40-CD40L interactions deliver the second, driving differentiation of T cells into effector T cells (CD25^+^ T cells) and/or Tregs (CD25^+^FoxP3^+^ T cells). Activated T cells express CTLA-4, which competes with CD28 for CD80/CD86, limiting excessive activation. B cells secrete IL-6 and IL-10, and some become Bregs producing IL-10. Bregs and Tregs form a loop maintaining pregnancy immune tolerance. (**B**) TLR9 blockade with ODN 2088 during early pregnancy decreased CD80 and increased CD86 on B cells, impairing antigen presentation and reducing activation of effector T cells and Tregs in pregnant females in the murine abortion-prone model. Red arrows indicate decreased expression; blue arrows indicate increased expression. Abbreviations: TLR9, Toll-like receptor 9; TCR, T-cell receptor; Tregs, regulatory T cells; Bregs, regulatory B cells; mtDNA, mitochondrial DNA; cffDNA, cell-free fetal DNA. The figure was created using BioRender (https://BioRender.com/dd94nle, accessed on 19 December 2025).

Taken together, this study demonstrated that blocking TLR9 signaling in CBA/J females shortly after mating with DBA/2J males increased pregnancy loss without affecting implantation success. We further showed that the administration of the TLR9 antagonist altered the costimulatory phenotype of both B and T cells, influencing the activation status of T cells and the development of Tregs in both pre-implantation and the more advanced stages of pregnancy. The proposed mechanism of TLR9 function, including its inhibition by ODN 2088 and the resulting effects on B and T cells, is summarized in Figure 6.

These findings indicate that, while TLR9 inhibition has been proposed as a potential therapeutic approach in immune-mediated pregnancy complications [16,17,21], peri-implantation blockade of this receptor using ODN 2088 worsened reproductive outcomes in an abortion-prone mouse model. The novelty of this study lies in the observation that TLR9 signaling, rather than being universally detrimental, may be required in the appropriate immunological context to maintain immune equilibrium at the early stages of gestation. These findings emphasize the need for caution when considering TLR9 inhibition during early pregnancy, especially in women with pregnancy disorders associated with excessive inflammatory activation. Importantly, to the best of our knowledge, this study is the first to investigate the effects of TLR9 inhibition during early pregnancy, thereby expanding the current understanding of how innate immune pathways contribute to successful pregnancy establishment.

## 4. Materials and Methods

### 4.1. Animals

All mice were purchased from Charles River Laboratories (France) and housed under specific pathogen-free (SPF) conditions in individually ventilated cages with a 12 h:12 h dark–light cycle. The reproductive cycle of CBA/J females was monitored by staining vaginal smears with Cytocolor (Merck Millipore, Darmstadt, Germany), according to the manufacturer’s instructions. Between 6:00 and 7:00 p.m., females in the proestrus phase were paired with DBA/2J males for mating. The following morning, between 7:00 and 8:00 a.m., CBA/J females were inspected for the presence of vaginal plugs, which indicated successful mating. This was designated as 0 dpc. Immediately after confirming the presence of copulating plugs, the females were intraperitoneally injected with either 50 µg or 100 µg of the TLR9 antagonist ODN 2088 (Invivogen, San Diego, CA, USA) (dissolved in 100 µL of PBS) or PBS (control group). The ODN 2088 doses (50 µg and 100 µg) were selected based on findings from earlier studies conducted by other researchers in pregnant females using NOD, IL-10^−/−^, and CBA/J × DBA/2J, mouse models, in which these doses effectively inhibited TLR9 activation and reduced the incidence of embryo resorption. In those studies, both doses demonstrated biological efficacy with no observed signs of toxicity in the female mice [22,25,27].

At 3 dpc (pre-implantation period of pregnancy) or 14 dpc (period of pregnancy when the placenta is fully developed and feto–maternal contact is stabilized), pregnant mice were euthanized by cervical dislocation, and the spleen, PALNs, and uterus were dissected. Pregnancy at 3 dpc was confirmed by checking uterine flushings under a microscope for the presence of embryos. At 14 dpc, pregnancy was confirmed by the presence of viable fetuses and resorbed embryos in the uterus. Resorbed embryos were identified based on their size and necrotic and hemorrhagic appearance. At 14 dpc, the abortion rate was calculated using the following formula [82]:

$$\frac{number\;of\;resorbed\;embryos}{total\;number\;of\;implantation\;sites\;(number\;of\;resorbed\;embryos\;+\;number\;of\;viable\;fetuses)}\times 100\%$$

**Figure 7:**
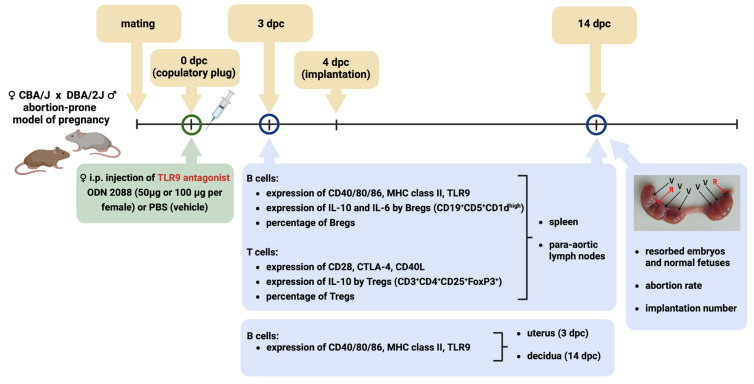
Schematic representation of the experimental design. Female CBA/J mice were mated with male DBA/2J mice (an abortion-prone model of pregnancy). After confirmation of mating, females were injected intraperitoneally with ODN 2088 (a TLR9 antagonist) or PBS (control). At 3 dpc (the day before implantation) and 14 dpc, flow cytometry was used to analyze the expression of costimulatory molecules (CD40, CD80, and CD86), MHC class II, and TLR9 on/in B cells derived from the spleen, para-aortic lymph nodes draining the uterus, uterus, and decidua. Additionally, T and B regulatory cells, cytokine expression, and the expression of receptors for costimulatory molecules (CD28, CTLA-4, and CD40L) on T cells derived from the spleen and para-aortic lymph nodes were analyzed. On day 14 of pregnancy, implantation sites were evaluated, and the numbers of viable fetuses and resorbed embryos were counted to determine the abortion rate. Abbreviations: dpc, days post-coitum; i.p., intraperitoneal injection; Bregs, regulatory B cells; Tregs, regulatory T cells; TLR9, Toll-like receptor 9; V, viable fetuses; R, resorbed embryos. The figure was created using BioRender (https://BioRender.com/qvfbigr, accessed on 19 December 2025).

In Figure 7, we present a schematic representation of the experiment.

### 4.2. Tissue Dissociation and Cell Isolation

The spleen and PALNs were pushed through a 40 µm cell strainer into a 0.84% ammonium chloride solution or PBS, respectively. Cell suspensions were centrifuged (300× *g*, 10 min), and the splenocytes were washed twice with PBS.

Uteri from mice at 3 dpc were dissected and cut thoroughly with surgical scissors in the presence of digestion medium [HBSS (Biowest, Nuaillé, France) supplemented with 30 µg/mL DNase I (Roche, Basel, Switzerland) and 0.26 WU/mL Liberase TM (Roche, Basel, Switzerland)]. The digested tissues were further processed as previously described [67].

At 14 dpc, the deciduae from two placentas of morphologically normal fetuses from one mouse were dissected according to the method described by Croy et al. [83]. The deciduae were pushed through a 40 μm cell strainer into a Petri dish with PBS and centrifuged (300× *g*, 10 min). The isolated cells from the two deciduae were pooled and treated as a single sample.

### 4.3. Flow Cytometry Staining

Cells isolated from the spleen and lymph nodes were cultured in RPMI-1640 medium (Biowest, Nuaillé, France) supplemented with 10% fetal bovine serum, 0.1 μg/mL phorbol 12-myristate 13-acetate (PMA; Cayman Chemical, Ann Arbor, MI, USA), 1 μg/mL ionomycin (Cayman Chemical, Ann Arbor, MI, USA), 2 μM monensin A (Biolegend, San Diego, CA, USA), and 10 μg/mL brefeldin A (Biolegend, San Diego, CA, USA) at 37 °C in 5% CO_2_ for 4 h. After washing, cell viability was assessed using the Zombie Red™ Fixable Viability Kit (Biolegend, San Diego, CA, USA), and Fc receptors were blocked with anti-mouse CD16/CD32 antibody (clone 93; Thermo Fisher Scientific, Waltham, MA, USA). Extracellular and intracellular staining were performed as previously described [67]. All antibodies were purchased from Biolegend (Biolegend, San Diego, CA, USA), unless otherwise stated. The following fluorochrome-conjugated antibodies were used for extracellular staining: anti-CD3 (clone 17A2), anti-CD4 (clone GK1.5), anti-CD25 (clone 3C7), anti-CD152 (clone UC10-4B9), anti-CD154 (clone MR1), anti-CD28 (clone 37.51; BD Biosciences, San Jose, CA, USA), anti-CD19 (clone 6D5), anti-CD5 (clone 53-7.3), anti-CD1d (clone 1B1), anti-CD86 (clone GL-1), anti-CD80 (clone 16-10A1), anti-CD40 (clone 3/23), anti-I-A/I-E (MHC class II; clone M5/114.15.2), and anti-TLR9 (clone S18025A), or the respective isotype controls.

Intracellular staining was performed using the True-Nuclear™ Transcription Factor Buffer Set (Biolegend, San Diego, CA, USA), according to the manufacturer’s instructions. The following fluorochrome-conjugated antibodies were used for intracellular staining: anti-FoxP3 (clone FJK-16s; Thermo Fisher Scientific, Waltham, MA, USA), anti-IL-10 (clone JES5-16E3), and anti-IL-6 (clone MP5-20F3).

To determine the expression of CD80, CD86, CD40, MHC class II, and TLR9 on/in B lymphocytes derived from the uterus and decidua, cells were stained in a manner analogous to those isolated from the spleen and lymph nodes, using the same reagents and antibodies. The only difference was additional staining with anti-CD45 (clone 30-F11).

The samples were immediately analyzed using an LSRFortessa™ flow cytometer (BD Biosciences, San Jose, CA, USA). All analyses were performed using the FlowJo™ v10.8.1 software (BD Biosciences, San Jose, CA, USA).

Figures S1 and S2 in the Supplementary Materials show the representative gating strategies for the cells of interest. The expression levels of the studied molecules and cytokines in T and B cells are expressed as median fluorescence intensity (MFI), calculated based on the difference between the MFI of the specifically stained cells and that of the isotype-matched control cells gated for the populations of interest.

### 4.4. Statistical Analysis

Comparisons of immune cell phenotypes and expression of selected molecules were performed only within the same gestational stages (3 dpc or 14 dpc), representing distinct phases of pregnancy, to maintain a consistent hormonal and immunological background and ensure that observed differences could be attributed specifically to TLR9 antagonist (ODN 2088) administration.

Statistical analyses were performed using the GraphPad Prism v5.03 software (GraphPad, San Diego, CA, USA). Normality was assessed using the Shapiro–Wilk normality test. When the data were normally distributed and variances were homogeneous, a one-way analysis of variance (ANOVA) was performed, followed by Dunnett’s multiple comparison post hoc test to compare the data between groups. The Kruskal–Wallis test followed by Dunn’s multiple comparison post hoc test was used when the data were not normally distributed or when the variances were not homogeneous between groups (according to Bartlett’s test). A *p*-value < 0.05 was considered statistically significant.

## 5. Conclusions

Blocking TLR9 signaling shortly after mating in the CBA/J × DBA/2J, abortion-prone model did not improve pregnancy outcomes but rather exacerbated pregnancy loss without affecting implantation success. The costimulatory phenotypes of B and T cells were concurrently altered, influencing T cell activation and impairing Treg development during both pre-implantation and the later stage of pregnancy.

## Data Availability

The data presented in this study are available on request from the corresponding author.

## Supplementary Materials

The following supporting information can be downloaded at https://www.mdpi.com/article/10.3390/ijms27020848/s1.

## Abbreviations

The following abbreviations are used in this manuscript:

ANOVA	analysis of variance
APCs	antigen-presenting cells
Bregs	regulatory B cells
cffDNA	cell-free fetal DNA
dpc	days post coitum
FGR	fetal growth restriction
IUGR	intrauterine growth restriction
MFI	median fluorescence intensity
mtDNA	mitochondrial DNA
NOD	non-obese diabetic
ODNs	oligodeoxynucleotides
PALNs	para-aortic lymph nodes
PBS	phosphate-buffered saline
PE	preeclampsia
PRRs	pattern recognition receptors
RPL	recurrent pregnancy loss
SPF	specific pathogen-free
TCR	T-cell receptor
TLRs	Toll-like receptors
Tregs	regulatory T cells

## Appendix A

**Table A1:** Summary of the expression of costimulatory molecules on B cells and the percentage of B cells expressing these molecules among B-cell populations in abortion-prone female mice.

		B Cells (CD19^+^)									
		CD80		CD86		CD40		MHC II		TLR9	
		MFI	%	MFI	%	MFI	%	MFI	%	MFI	%
3 dpc 50 µg ODN 2088	lymph nodes	↓ *	-	↑ *	-	-	-	-	-	-	-
	spleen	-	-	-	-	-	-	-	-	-	-
	uterus	-	-	-	-	-	-	-	-	-	-
3 dpc 100 µg ODN 2088	lymph nodes	-	-	-	-	-	-	-	-	-	-
	spleen	-	-	-	-	-	-	-	-	-	-
	uterus	-	-	-	-	-	-	-	-	-	-
14 dpc 50 µg ODN 2088	lymph nodes	-	-	-	-	-	-	-	-	-	-
	spleen	-	↓ *	-	-	-	-	-	-	-	-
	decidua	-	-	-	-	-	-	-	-	↓ *	-
14 dpc 100 µg ODN 2088	lymph nodes	↓ *	-	-	-	-	-	-	-	↓ *	↓ *
	spleen	-	↓ *	-	-	-	-	-	-	↓ *	↓ *
	decidua	-	-	-	-	-	-	-	-	↓ ***	↓ *

The data show changes in the MFI of CD80, CD86, CD40, MHC class II, and TLR9 on/in B cells, as well as the percentage of B cells expressing these molecules, among B-cell populations derived from the spleen, para-aortic lymph nodes draining the uterus, uterus (3 dpc), or decidua (14 dpc) of abortion-prone female mice after intraperitoneal administration of the TLR9 antagonist ODN 2088. Statistical analyses were performed using one-way ANOVA with Dunnett’s multiple comparison post hoc test (for normally distributed data) or the Kruskal–Wallis test with Dunn’s multiple comparison post hoc test (for non-normally distributed data or those with non-equal variances). Normality was assessed using the Shapiro–Wilk test, and Bartlett’s test was performed to verify the homogeneity of variances between groups. * *p* < 0.05; *** *p* < 0.001. Legend: dpc, days post-coitum; Bregs, regulatory B cells; Tregs, regulatory T cells; MFI, median fluorescence intensity; ↑, increased MFI/percentage compared to control mice; ↓, decreased MFI/percentage compared to control mice; -, no change in MFI/percentage compared to control mice.

**Table A2:** Summary of the expression of ligands for costimulatory molecules on T cells and the percentage of T cells expressing these ligands among T cells in abortion-prone female mice.

		T Cells (CD3^+^CD4^+^)					
		CD28		CTLA-4		CD40L	
		MFI	%	MFI	%	MFI	%
3 dpc 50 µg ODN 2088	lymph nodes	-	-	-	-	↓ ***	-
	spleen	-	-	-	-	↓ **	-
3 dpc 100 µg ODN 2088	lymph nodes	-	-	-	-	↓ **	-
	spleen	-	-	-	-	-	-
14 dpc 50 µg ODN 2088	lymph nodes	-	-	-	-	-	-
	spleen	↓ ***	↓ *	-	-	-	-
14 dpc 100 µg ODN 2088	lymph nodes	-	-	-	-	-	-
	spleen	-	-	-	-	-	-

The data show changes in the MFI of CD28, CTLA-4, and CD40L on T cells, as well as the percentage of T cells expressing these molecules among T-cell populations derived from the spleen and para-aortic lymph nodes draining the uterus of abortion-prone female mice following intraperitoneal administration of the TLR9 antagonist ODN 2088. Statistical analyses were performed using one-way ANOVA with Dunnett’s multiple comparison post hoc test (for normally distributed data) or the Kruskal–Wallis test with Dunn’s multiple comparison post hoc test (for non-normally distributed data or those with non-equal variances). Normality was assessed using the Shapiro–Wilk test, and Bartlett’s test was performed to verify the homogeneity of variances between groups. * *p* < 0.05; ** *p* < 0.01; *** *p* < 0.001. Legend: dpc, days post-coitum; Bregs, regulatory B cells; Tregs, regulatory T cells; MFI, median fluorescence intensity; ↓, decreased MFI/percentage compared to control mice; -, no change in MFI/percentage compared to control mice.

**Table A3:** Summary of the percentages of CD3^+^CD4^+^CD25^+^ cells, Tregs, and Bregs, as well as the expression levels of IL-10 in Bregs and Tregs, and IL-6 in Bregs from abortion-prone female mice.

		CD3^+^CD4^+^CD25^+^ Cells	Tregs: CD3^+^CD4^+^CD25^+^FoxP3^+^		Bregs: CD19^+^CD5^+^CD1d^high^		
		% Among CD3^+^CD4^+^	% Among CD3^+^CD4^+^	IL-10 Expression (MFI)	% Among CD19^+^	IL-10 Expression (MFI)	IL-6 Expression (MFI)
3 dpc 50 µg ODN 2088	lymph nodes	-	-	-	-	-	-
	spleen	-	↓ *	-	-	-	-
3 dpc 100 µg ODN 2088	lymph nodes	-	-	-	-	-	-
	spleen	-	-	-	-	-	-
14 dpc 50 µg ODN 2088	lymph nodes	↓ **	↓ *	-	-	-	-
	spleen	↓ *	-	-	-	-	-
14 dpc 100 µg ODN 2088	lymph nodes	-	-	-	-	-	-
	spleen	-	-	-	-	-	-

The data show changes in the frequencies of Breg, Treg, and activated T cells, as well as the expression of IL-10 and IL-6 by Bregs and IL-10 by Tregs in the spleen and para-aortic lymph nodes following intraperitoneal administration of the TLR9 antagonist ODN 2088. Statistical analyses were performed using one-way ANOVA with Dunnett’s multiple comparison post hoc test (for normally distributed data) or the Kruskal–Wallis test with Dunn’s multiple comparison post hoc test (for non-normally distributed data or those with non-equal variances). Normality was assessed using the Shapiro–Wilk test, and Bartlett’s test was performed to verify the homogeneity of variances between groups. * *p* < 0.05; ** *p* < 0.01. Legend: dpc, days post-coitum; Bregs, regulatory B cells; Tregs, regulatory T cells; MFI, median fluorescence intensity; ↓, decreased MFI/percentage compared to control mice; -, no change in MFI/percentage compared to control mice.
