# Supplementary material for: TLR9 Inhibition Shortly After Mating Increases Fetal Resorption and Alters B- and T-Cell Costimulatory Phenotypes in an Abortion-Prone Mouse Model
Source: Int J Mol Sci. 2026 Jan 14;27(2):848. doi: 10.3390/ijms27020848 (PMC12840859; doi:10.3390/ijms27020848)
Supplement: Supplementary file 1 [file ijms-27-00848-s001.zip › Supplementary materials.pdf]

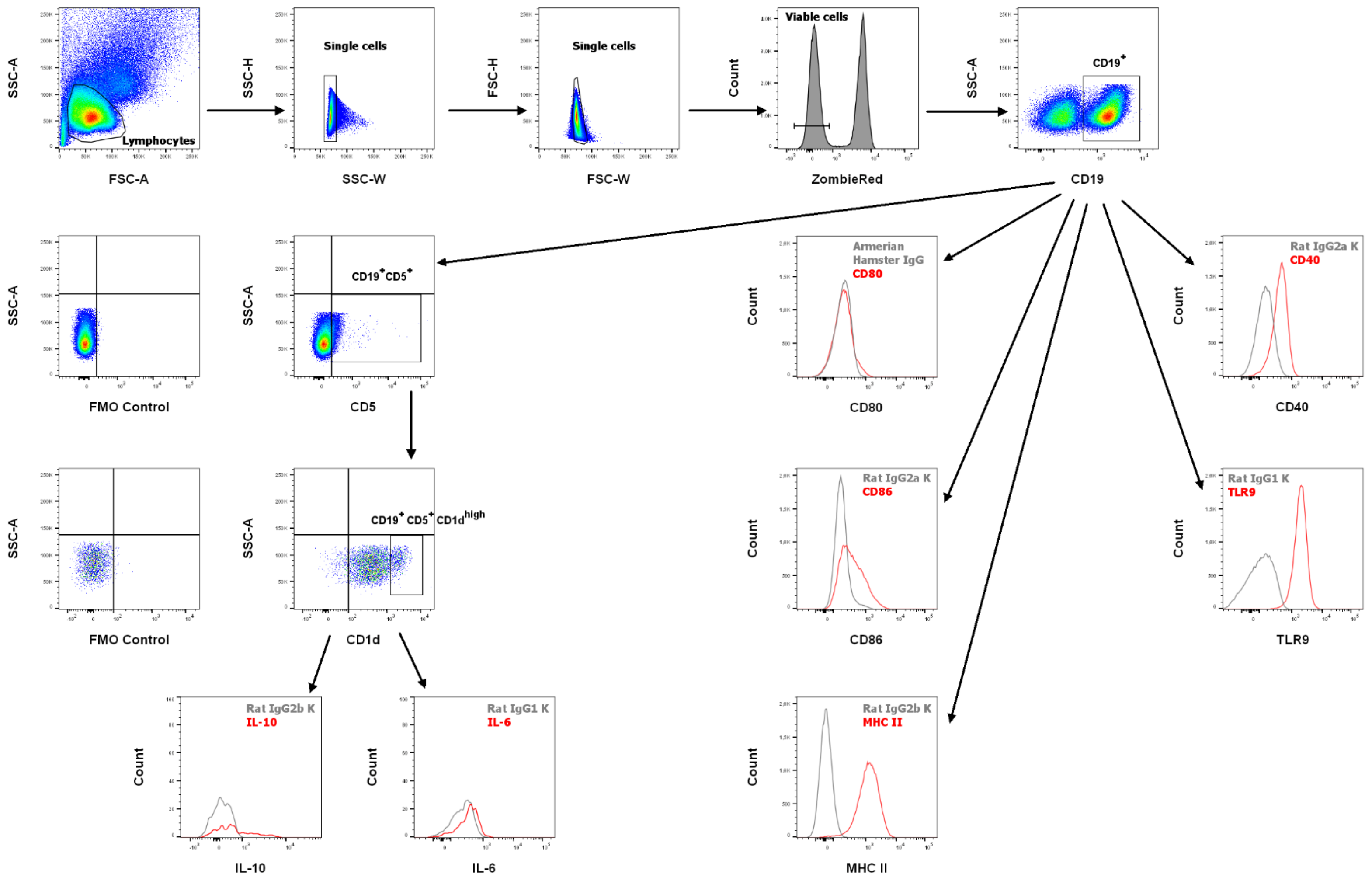


**Supplementary Figure S1. Representative gating strategy for regulatory B cells and assessment of cytokine and costimulatory molecule expression by B cells.** Representative dot plots illustrating the gating strategy for Bregs (CD19$^{+}$CD5$^{+}$CD1d$^{high}$) and overlay histograms comparing the expression of IL-10, IL-6, CD80, CD86, CD40, MHC class II, and TLR9 (red) with their respective isotype-matched controls (gray) in B cells (or in Bregs, in the case of IL-10 and IL-6). Data obtained from the splenocytes of pregnant CBA/J mice.

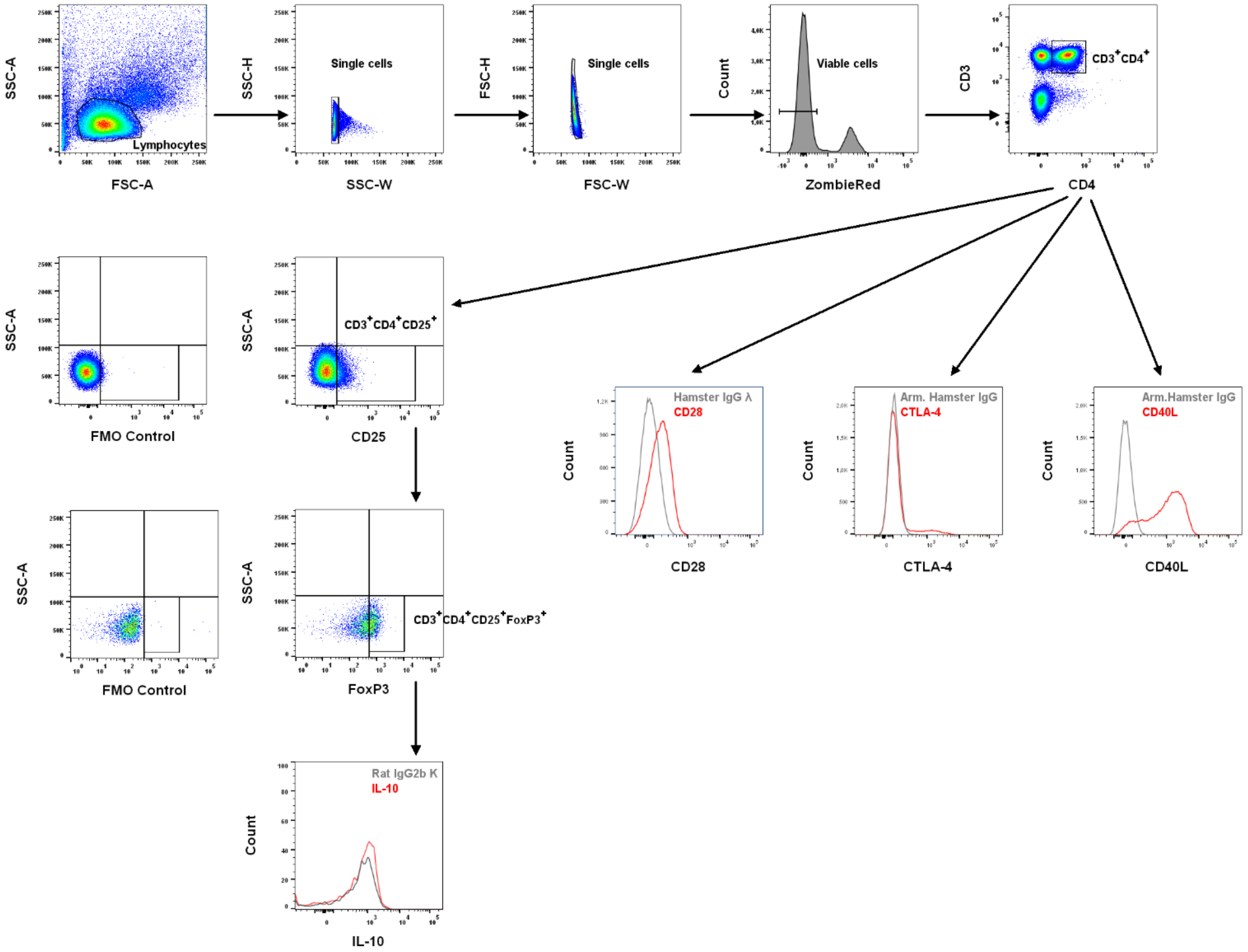


**Supplementary Figure S2. Representative gating strategy for T cell subsets and expression of cytokines and ligands for costimulatory molecules by T cells.** Representative dot plots illustrating the gating strategy for T cells ($CD3^+CD4^+$), activated T cells ($CD3^+CD4^+CD25^+$), and Tregs ($CD3^+CD4^+CD25^+FoxP3^+$), with overlay histograms comparing the expression levels of IL-10, CD28, CTLA-4, and CD40L (red) with their respective isotype-matched controls (gray) in T cells (or in Tregs, in the case of IL-10). Data obtained from the uterine-draining lymph nodes of pregnant CBA/J mice.
