# Supplementary figures and images for: TLR9 Inhibition Shortly After Mating Increases Fetal Resorption and Alters B- and T-Cell Costimulatory Phenotypes in an Abortion-Prone Mouse Model
Source: Int J Mol Sci. 2026 Jan 14;27(2):848. doi: 10.3390/ijms27020848 (PMC12840859; doi:10.3390/ijms27020848)

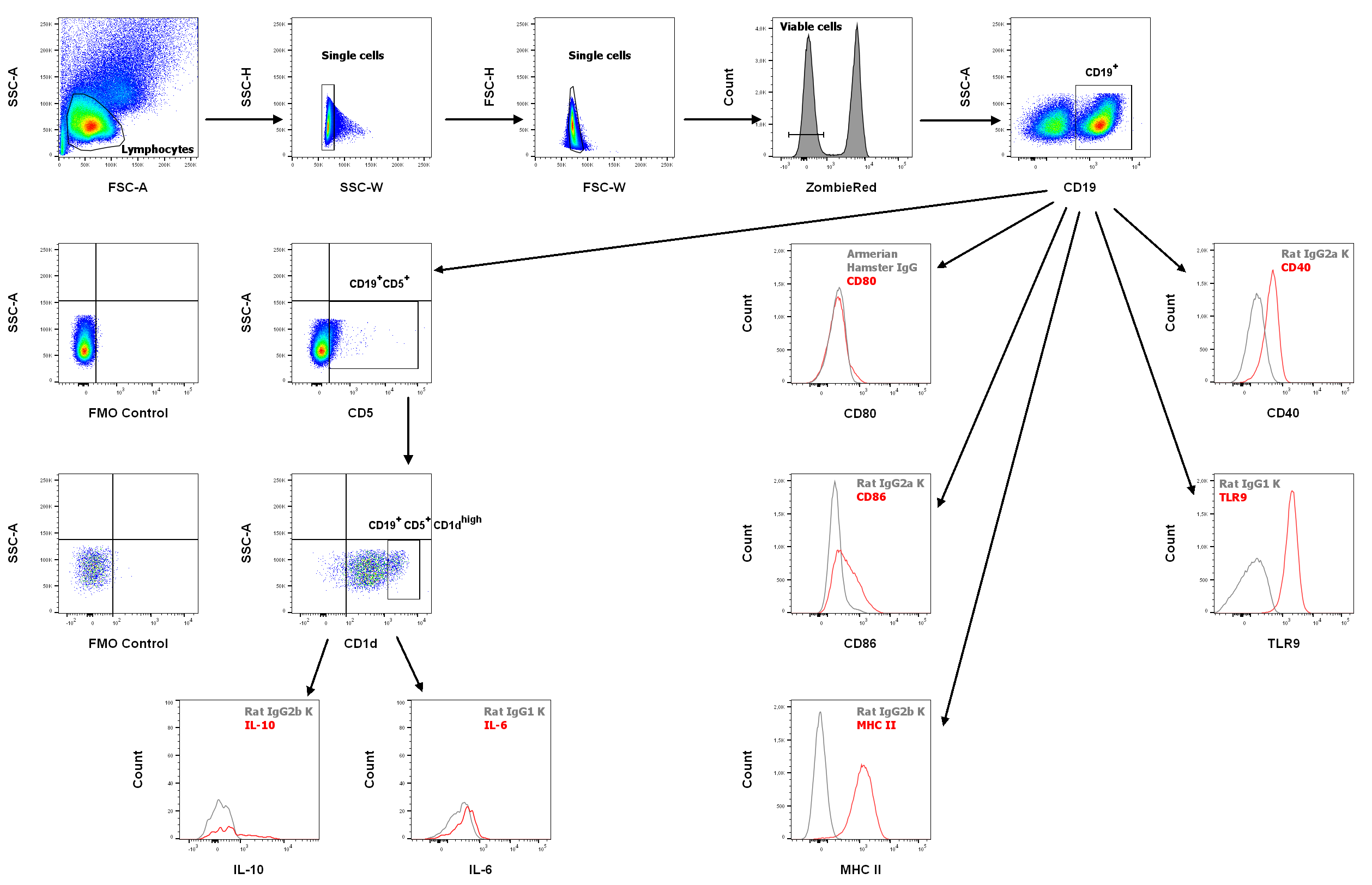

Supplement: Supplementary file 1 [file ijms-27-00848-s001.zip › Supplementary Figure_1.tiff]

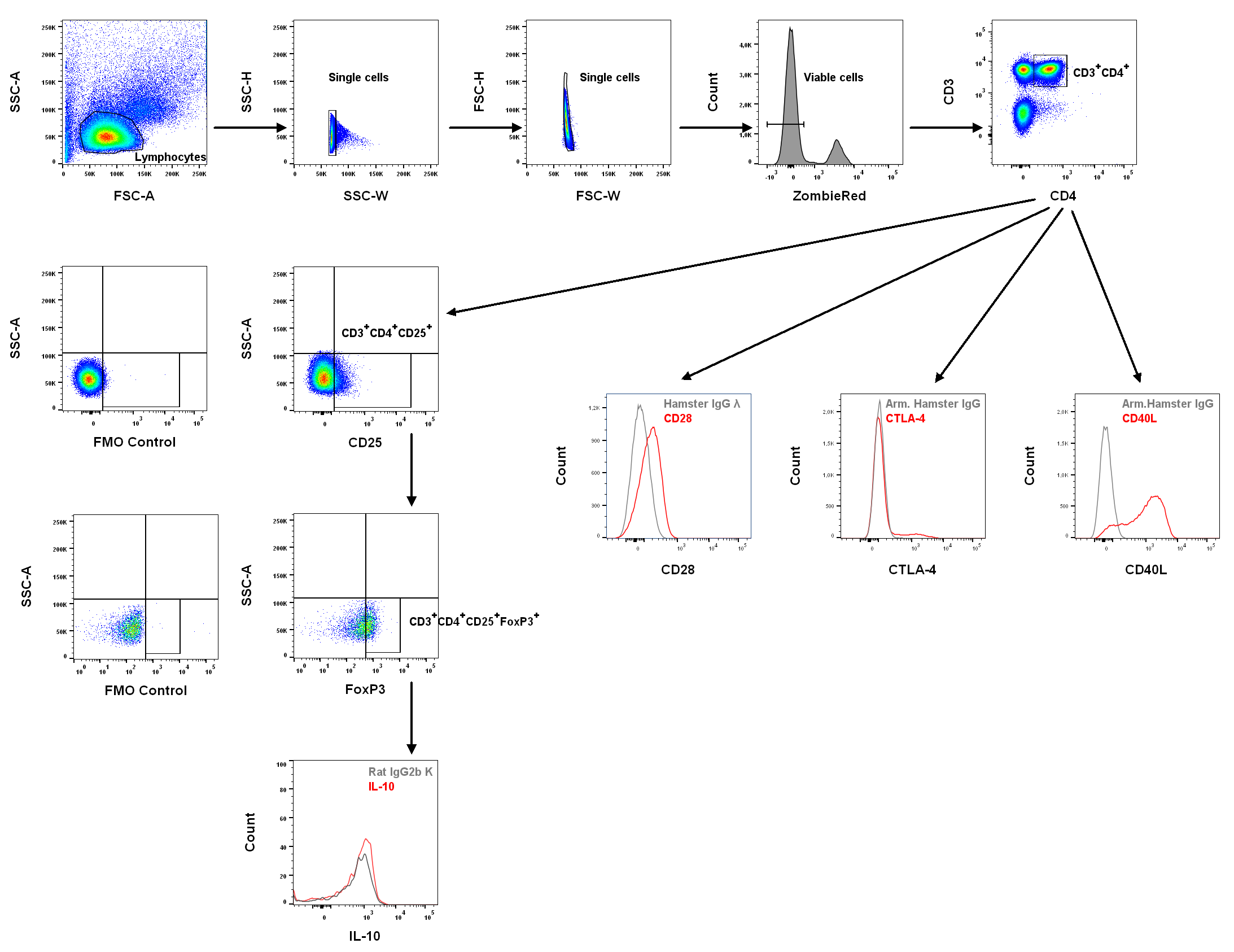

Supplement: Supplementary file 1 [file ijms-27-00848-s001.zip › Supplementary Figure_2.tiff]
